# Intestinal Obstruction Secondary to Multiple Gastrointestinal phytobezoars, A Rare presentation

**DOI:** 10.1016/j.ijscr.2021.106004

**Published:** 2021-05-21

**Authors:** Abdullah G. Alsahwan, Ameen M. Almarhoon, Jihad AlSafwani, Hanan Alsahwan, Neamat Alturki

**Affiliations:** a Department of General Surgery, Qatif Central Hospital, Qatif, Saudi Arabia; b Imam Abdulrahman Bin Faisal University, Dammam, Saudi Arabia

**Keywords:** Bezoar, Phytobezoars, Persimmon, Gastrostomy, Enterotomy

## Abstract

**Introduction:**

Intestinal obstruction considered to be one of the most common surgical presentation. Adhesions secondary to previous operations, hernias, neoplasms, inflammatory bowel disease, intussusception, or volvulus are the usual causes of intestinal obstruction but bezoar can presents in 0.4-4%. Bezoar can be trapped in different locations throughout the gastrointestinal tract and it can be solitary or multiple lesions.

**Case presentation:**

This is a 37-year-old male, known case of diabetes mellitus, Presented to the Emergency Department complaining of generalized abdominal pain for 2 days duration. Associated with abdominal distention, fever, nausea, vomiting and obstipation. There was a history of persimmon intake. Unremarkable past surgical history. On examination, He was tachycardic, other vital signs were within normal. Abdominal examination showed abdominal distention and Sluggish bowel sound. Abdominal X-ray revealed multiple air-fluid levels. An abdominal CT scan with IV contrast revealed an intra-luminal mass in the ileum and intra-gastric mass with suspicious of bezoars. He underwent exploratory laparotomy, gastrostomy to remove intra-gastric bezoar, and enterotomy to remove the ileal bezoar.

**Clinical discussion:**

Intestinal obstruction is considered to be the most common complication of this entity; other possible complications include gastric ulcer, gastritis, and gastric perforation. Due to limitations of endoscopy and barium enema in the diagnosis of bezoar, Abdominal CT-scan is considered to be the gold standard in the diagnosis. The management of phytobezoar can be either conservative or surgical, depends on the lesion size and location.

**Conclusion:**

Although intestinal obstruction secondary to bezoar is rare, multiple levels of gastrointestinal obstruction should raise the suspicion of bezoar.

## Highlights

• Bezoar can be trapped in different locations throughout the gastrointestinal tract and it can be solitary or multiple lesions.
• The predisposing risk factors for bezoar formation include previous gastric surgery, gastroparesis, peptic ulcer disease, or psychiatric illness.
• Abdominal CT-scan considered the gold standard in the diagnosis for bezoar.
• The management of phytobezoar can either be conservative or surgical depends on the lesion size and location.

## 1 Introduction

Intestinal obstruction considered to be one of the most common surgical presentation, adhesions secondary to previous operations, hernias, neoplasms, inflammatory bowel disease, intussusception, or volvulus are the usual causes of intestinal obstruction but bezoar can present in 0. 4–4%. Bezoar can be trapped in different locations throughout the gastrointestinal tract and it can be solitary or multiple lesions. We are reporting a case of an adult male with intestinal obstruction secondary to multiple gastrointestinal phytobezoars, managed surgically.

The work has been reported in line with the SCARE 2020 criteria [11].

## 2 Case presentation

This is a 37-year-old male, known case of diabetes mellitus, Presented to the Emergency Department complaining of Generalized abdominal pain for 2 days duration. Associated with abdominal distension, fever, nausea, vomiting and obstipation. He denied any history of eating from outside, but he gave a history of persimmon intake. Unremarkable past surgical history. His family, drug and psychological history were unremarkable. On examination, He was tachycardic, other vital signs were within normal. Abdominal examination showed abdominal distention and Sluggish bowel sound. Digital rectal examination showed an empty rectum. Laboratory investigations showed leukocytosis 16 thousands per cubic milliliter, other labs were unremarkable. Abdominal X-ray revealed multiple air-fluid levels. Abdominal CT scan with IV contrast revealed dilated small bowel loops reaching up to 3.8 cm with intraluminal mass in the ileum measuring 3.4 × 2.2 × 3.3 cm (Fig. 1) and intra-gastric mass measuring 3 × 7 × 3 cm (Photo 2, Photo 3) with bezoars suspicion. After resuscitation, he underwent a trial to remove the intra-gastric bezoar by upper endoscopy, a small part of the bezoar removed. After that, he underwent exploratory laparotomy, gastrostomy to remove intra-gastric bezoar (Fig. 4), and enterotomy to remove the ileal bezoar (Photo 5, Photo 6) by a general surgeon. The histopathological examination of the bezoars revealed vegetable materials with inflammatory cells. Eventually, he was discharged home in a good condition.

**Photo 1:**
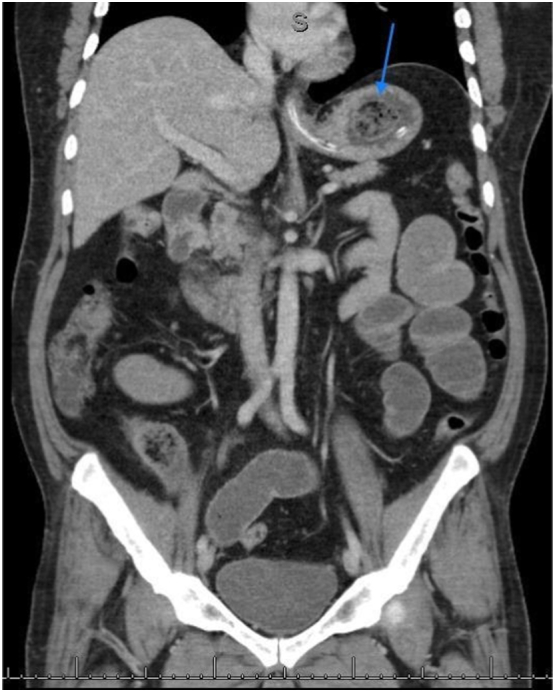
A sagittal plane of CT abdomen showing a large intra-gastric mass, pointed by the blue arrow.

**Photo 2:**
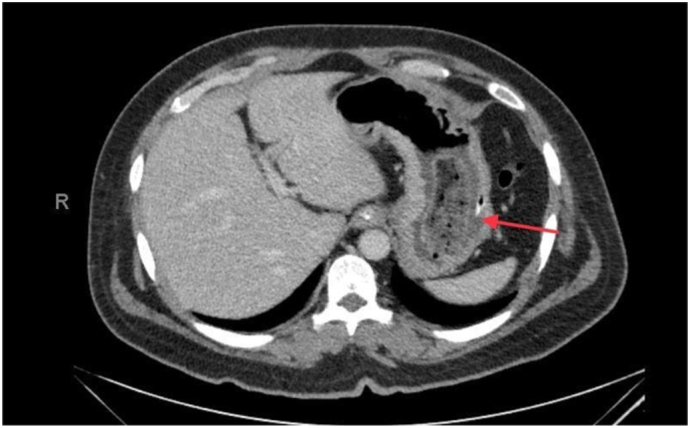
A axial plane of CT abdomen showing a large intra-gastric mass, pointed by the red arrow.

**Photo 3:**
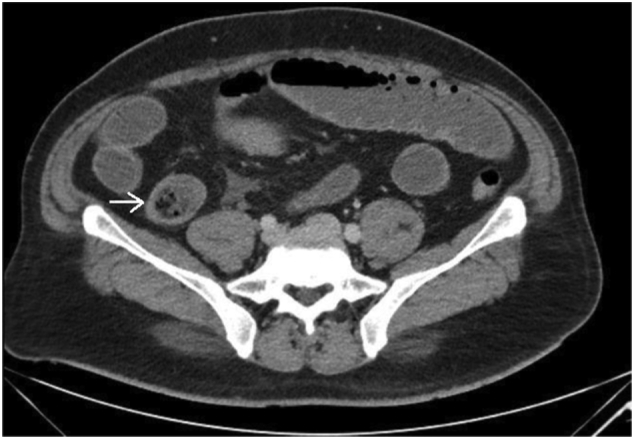
A axial plane of CT abdomen showing an intraluminal mass in the ileum, pointed by the white arrow.

**Photo 4:**
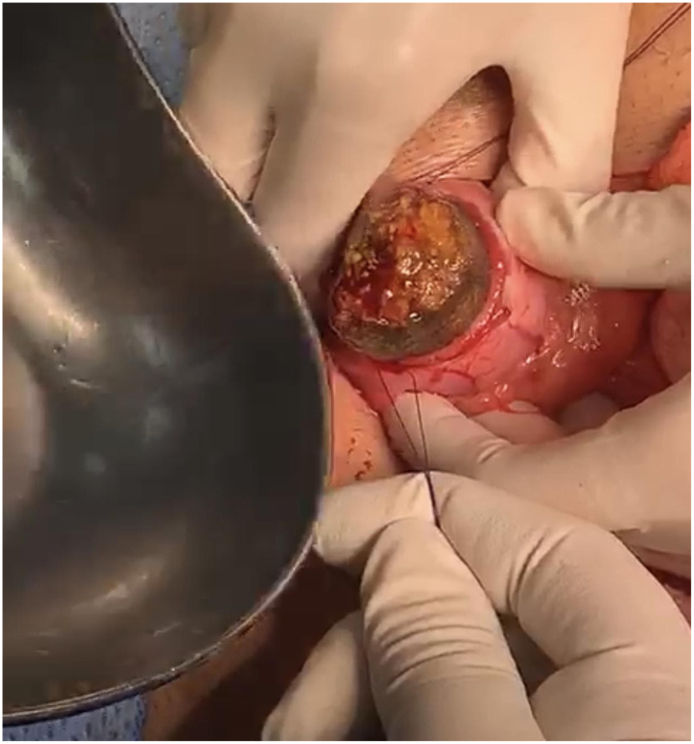
Intra-operative removal of the gastric bezoar by gastrostomy.

**Photo 5:**
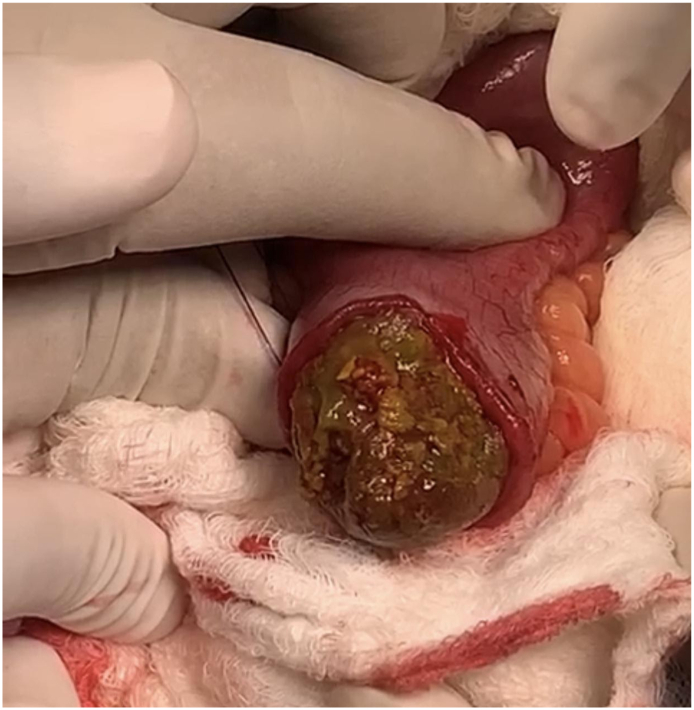
Intra-operative removal of the ileal bezoar by ileostomy.

**Photo 6:**
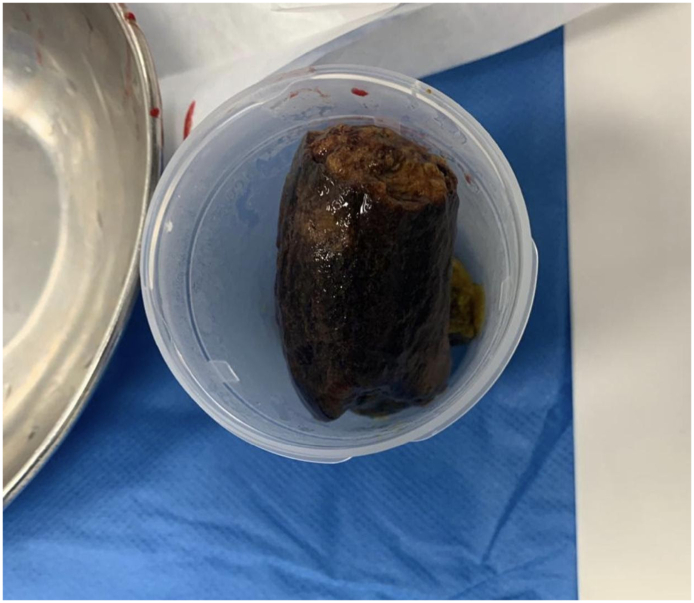
A phytobezoar.

## 3 Discussion

Bezoar is a rare entity, usually defined as a mass formed by undigested foreign materials that accumulated and trapped inside the gastrointestinal tract [1]. There are different types of bezoars depend on their material composition, the most common type of bezoar is phytobezoar (vegetable fibers), other types are trichobezoar (hair), pharmacobezoar (medications), lactobezoar (milk remnants), and lithibezoar (stones) [2,3].

The predisposing risk factors for bezoar formation include pyloric dysfunction, decrease gastric motility and decrease acid secretion secondary to previous gastric surgery, peptic ulcer disease, pyloric stenosis, gastrointestinal amyloidosis, poor mastication, dental implants, inadequate fluid intake, high amounts of indigestible fibers, psychiatric illness, or gastroparesis secondary to diabetes mellitus, hypothyroidism, or connective tissue diseases **[**4]. Erzurumlu et al. as reported in their retrospective study of 34 cases of bezoars, the most common predisposing factors are previous gastric surgery i.e. (Trancal vagotomy+pyloroplasty, distal subtotal gastrectomy or truncal vagotomy + billroth II anastomosis) accounts for 55.88%, followed by persimmon intake, diabetes mellitus, and peptic ulcer disease which account for 17.64%, 11.76%, and 11.76% respectively [5].

Usually, the patients present with abdominal pain, epigastric fullness, nausea, vomiting, dyspepsia, anorexia, or symptoms of bowel obstruction [5]. The incidence of gastrointestinal obstruction secondary to bezoar is 0.4–4% [6]. Intestinal obstruction is considered to be the most common complication of this entity; other possible complications include gastric ulcer, gastritis, and gastric perforation [7].

Due to limitations of endoscopy and barium enema in the diagnosis of bezoar in an emergency sitting which can worse symptoms in complete bowel obstruction, increase risk perforation, and worse peritonitis in the presence of perforation. Abdominal CT-scan is considered to be the gold standard in the diagnosis with a sensitivity of 90% and a specificity of 57% [1]. In a study of 39 patients with 54 gastrointestinal phytobezoars evaluated by CT-scan, the location of the bezoars were (21.4% in the Stomach, 1.7% in the duodenum, 46.4% in the jejunum, and 30.3% in the ileum), Solitary bezoar was found in (59% of the patients and multiple were found in 41%), the locations for multiple bezoars were (15.3% in the stomach and jejunum, 12.8% in the stomach and ileum, 5.1% in the jejunum and ileum, 2.5% in the stomach and multiple bezoars in the jejunum and 5.1% with multiple Jejunal bezoars) [8].

The management of phytobezoar can either be conservative, endoscopic or surgical depends on the lesion size and location. The conservative options may include dissolution by Coca-Cola, papain, L-cysteine, metoclopramide, cellulose, pineapple juice, sodium bicarbonate, hydrochloric acid, pancrelipase, pancreatin or 1–2% zinc chloride. Endoscopic option is considered in the removal of small-size gastric bezoar. Surgical options include laparoscopic or open removal, which is considered for large-size gastric or small bowel bezoar refractory to conservative management, or patients presenting with emergency conditions i.e. (bleeding or peritonitis). During surgical intervention, some surgeons prefer to mobilize the bezoar distal to the ileocecal valve using milking technique but this technique may cause serosal, mesenteric laceration or distal obstruction [9], other surgical options include enterotomy with primary closure, segmental resection with anastomosis in case of perforation or bowel ischemia [10].

## 4 Conclusions

Although intestinal obstruction secondary to bezoar is rare, multiple levels of gastrointestinal obstruction should raise the suspicion of bezoar.

## Funding

This study did not receive any funding.

## Ethical approval

IRB approval is not needed for Case reports in our center.

## Consent

Written informed consent was obtained from the patients for publication of this case report, A copy of the written consent is available for review by the Editor-in-Chief of this journal on request.

## Registration of research studies

Not applicable.

## Guarantor

Dr. Neamat Alturki.

Dr. Abdullah G. AlSahwan.

## Provenance and peer review

Not commissioned, externally peer-review.

## CRediT authorship contribution statement

Abdullah G. Alsahwan: Main Author of the paper, study concept and design, data collection, data interpretation, literature review, drafting of the paper, final review of the manuscript.

Ameen Almarhoon, Jihad Alsafwani, Hanan Alsahwan: study concept and design, data collection, data interpretation, literature review, drafting of the paper, final review of the manuscript.

Neamat Alturki: Supervisor; Treating physicians of the patient, study concept and design, data collection, data interpretation, literature review, drafting of the paper, final review of the manuscript.

## Declaration of competing interest

No declarations of interest.
